# A Dominant Negative Antisense Approach Targeting β-Catenin

**DOI:** 10.1007/s12033-018-0058-7

**Published:** 2018-03-09

**Authors:** Matthias Vonbrüll, Elisabeth Riegel, Christian Halter, Michaela Aigner, Holger Bock, Birgit Werner, Thomas Lindhorst, Thomas Czerny

**Affiliations:** 1 Department of Applied Life Sciences, University of Applied Sciences, FH Campus Wien, Helmut-Qualtinger-Gasse 2, 1030 Vienna, Austria; 2 Department of Engineering, University of Applied Sciences, FH Campus Wien, Favoritenstrasse 226, 1100 Vienna, Austria; 3 Present Address: Sandoz GmbH, Biochemiestraße 10, 6250 Kundl, Austria; 4 Present Address: CAST Gründungszentrum GmbH, Wilhelm-Greil-Straße 15, 6020 Innsbruck, Austria; 5 Present Address: UGISense AG, c/o Nordwind Capital GmbH, Residenzstrasse 18, 80333 Munich, Germany

**Keywords:** Antisense, β-Catenin, Morpholino, PNA, Wnt signalling

## Abstract

There have been many attempts to unveil the therapeutic potential of antisense molecules during the last decade. Due to its specific role in canonical Wnt signalling, β-catenin is a potential target for an antisense-based antitumour therapy. In order to establish such a strategy with peptide nucleic acids, we developed a reporter assay for quantification of antisense effects. The luciferase-based assay detects splice blocking with high sensitivity. Using this assay, we show that the splice donor of exon 13 of β-catenin is particularly suitable for an antisense strategy, as it results in a truncated protein which lacks transactivating functions. Since the truncated proteins retain the interactions with Tcf/Lef proteins, they act in a dominant negative fashion competing with wild-type proteins and thus blocking the transcriptional activity of β-catenin. Furthermore, we show that the truncation does not interfere with binding of cadherin and α-catenin, both essential for its function in cell adhesion. Therefore, the antisense strategy blocks Wnt signalling with high efficiency but retains other important functions of β-catenin.

**Electronic supplementary material:**

The online version of this article (10.1007/s12033-018-0058-7) contains supplementary material, which is available to authorized users.

## Introduction

In 1978 a synthetic antisense oligonucleotide was successfully used by Zamecnik and Stephensen for targeting of Rous sarcoma virus RNA within chick embryo fibroblasts [1]. Subsequently, a vast variety of new reagents were developed and tested [2], peaking in the FDA approval of Fomivirsen [3], Pegaptanib [4] and Mipomersen [5]. The successful clinical application demonstrated the potential of antisense reagents and motivated scientists to overcome issues with solubility, degradation or cellular delivery [6].

Due to their good affinity for complementary RNA sequences, lack of interactions with natural proteins and excellent water solubility, morpholino oligos became the gold standard for antisense knock-down experiments [7, 8]. They show a characteristic structure with a 6-membered morpholine ring replacing ring sugars present within naturally occurring nucleic acids. Furthermore, negatively charged phosphate linkages of DNA and RNA have been replaced by phosphorodiamidate intersubunit linkages [7].

In year 1991 peptide nucleic acids (PNAs) were developed by Nielsen and colleagues [9]. PNAs contain an artificial backbone made of N-(2-aminoethyl) glycine subunits. The nucleobases are attached to the α-amino group by an acetic acid linker [10]. Lack of negatively charged phosphate residues provides reduced electrostatic repulsion between the PNA strand and a target RNA or DNA strand, causing exceptional thermal stability of the resulting hybrid complexes [9]. In order to improve solubility as well as intracellular delivery, a variety of modifications have been described [11, 12]. Phosphonic ester-modified PNAs (pePNAs) represent a specific modification of the α-carbon atom of the glycine and improve the solubility and binding strength of interactions [13, 14].

The evolutionary highly conserved canonical Wnt pathway has shown to be crucial in development and disease [15]. Without binding of Wnt ligand proteins to frizzled/LRP5/6 co-receptors, β-catenin is recognised by a destruction complex including APC, Axin and GSK3β. As a result, β-catenin is degraded. In the active state the destruction complex is not formed; β-catenin accumulates and enters the nucleus where it interacts with members of the Tcf/Lef transcription factor family, such as Tcf3 and Lef1 [16]. During this process, Groucho/TLE proteins are replaced by β-catenin and co-activator proteins are recruited resulting in the activation of target genes [17, 18]. Besides its major role in Wnt signalling, β-catenin is also important for cell adhesion, where it connects cadherin to the cytoskeleton through α-catenin binding [19, 20].

Several components of the Wnt pathway, including APC, Axin, Tcf and β-catenin, have shown mutations relevant for human cancers [21, 22]. β-catenin mutations have been found in human colorectal cancer [23] and human hepatocellular carcinoma [24]. They result in constitutively active Wnt signalling, by uncoupling β-catenin from the degradation pathway [25, 26]. Due to its prominent role in the Wnt pathway, β-catenin has been discussed as a target for therapeutic applications [27, 28]. Targeting β-catenin showed a potential therapeutic benefit in a variety of studies, applying antisense reagents [27], peptides [29] or small molecule drugs [30].

In this study, we show a newly designed splice-based reporter assay for quantification of antisense effects, which allows a positive luminescence read out for successful splice blocking by antisense oligos. Based on this assay, a dominant negative knock-down strategy was developed for β-catenin with specific PNA and morpholino oligos. For this purpose, β-catenin was targeted in a way that the interaction with its main partners Tcf/Lef is still possible; however, interaction with essential coactivators is blocked.

## Materials and Methods

### Plasmids

For the splice-based reporter assay, a backbone containing a human EF1α short promoter [31] driving reporter gene expression, piggybac terminal repeat sequences for genome integration [32], puromycin resistance and a luciferase internal reference was constructed. Firefly luciferase (Fluc) [33] was used as reporter gene for pSplice2basis1 together with Gaussia luciferase (Gluc) [34] as an internal reference or NanoLuc (Nluc, Promega) [35] for pSplice3basis1 combined with the internal reference Fluc. Internal references were used for normalisation in order to compensate for varying cell number and viability. The consensus SD sequence of pSplice2basis1 and pSplice3basis1 is AGGTAAGT. β-catenin deletion constructs were generated via PCR and integrated into a pKC backbone containing a CMV promoter [36]. Mammalian two-hybrid constructs were all based on the pMC vector [37]. For details see Table S1.

### Cell Culture and Statistical Analysis

HeLa, HEK293 T-REx (Invitrogen) as well as SW480 cells were cultured in Dulbecco’s modified Eagle’s medium (DMEM/GE Healthcare) including 10% foetal bovine serum (Thermo Scientific) and 1% penicillin/streptomycin (Thermo Scientific) in a humidified environment with 5% CO_2_. Stable cell lines were generated using the piggybac transposon system [32]. Positive clones were picked after puromycin selection (Santa Cruz 1 µg/mL). Quantification of gels and Western blots was performed with ImageJ. *P* values were calculated by the Student’s *t* test. Statistical significance: *p ≤ 0.05; **p ≤ 0.01; ***p ≤ 0.001.

### Antisense Molecules

The synthesis of the lysine–phosphonic–ester-modified PNAs was performed as described in Jung et al. [14] by ugichem GmbH. Phosphorodiamidate morpholino oligonucleotides (abbreviated here as morpholinos) were manufactured by Gene Tools. All antisense molecules are listed in Table S2 with PNAs showing N-terminal (4-(tri-Fluoro-methyl)-phenyl)-acetyl-glycine (phenylacetate-glycine) modifications. The PNAs were dissolved in nuclease free water to 1 mM by repeated shaking and vortexing. Finally, they were gently sonicated for 2 min with repeated pulses. Subsequently, the PNAs were kept at − 80 °C.

### Transfection, Luciferase Assay and Mammalian Two-Hybrid Assay

For transient transfection cells were seeded in a PEI-coated [38] 96-well plate at a density of 0.5 × 10^4^ cells/well and grown over night (HeLa) or 48 h (HEK293 T-REx). Transfection was performed using TurboFect (Thermo Scientific) transfection reagent following instructions of the manufacturer. Measurement was performed as described [39]. For the mammalian two-hybrid experiments, 80 ng reporter plasmid (plucF24ZF), 2 ng reference (pMcGlucS) and 2 ng bait construct (see Table S1) were co-transfected per well with 22 ng truncated β-catenin constructs (prey) into HEK293 T-REx cells.

### Scraping

Cells were seeded in a 24-well plate with 0.3 × 10^5^ cells/well and incubated overnight. 24 h later medium was removed and 30 µL loading solution [40] including the antisense reagent (morpholino or PNA) as well as 0.1 mg/mL fluorescein–isothiocyanate–dextran (FITC-dextran FD10S, Sigma-Aldrich) was added. Subsequently, cells were scraped off the plate with a rubber policeman 5× clockwise and 5× counter clockwise, and remaining cells were washed off with 500 µL DMEM including 10% foetal bovine serum and 1% penicillin/streptomycin (Thermo Scientific) and transferred into a fresh 24-well plate. Cells were incubated 24 h for recovery until RNA extraction or luciferase measurements were performed.

### Electroporation

Cells were trypsinised, counted and diluted in DMEM to 3 × 10^7^ cells/mL and 30 µL transferred to a 1 mm gap electroporation cuvette (VWR) pre-cooled on ice. Adjustments for electroporation (device described in [41]) on the Accupulser were: pulse width 12 ms, pulse interval 20 ms, train duration 250 ms for HeLa or 500 ms for SW480, voltage 60 V at a frequency of 1 kHz. After electroporation, cells were transferred to a fresh pre-warmed 24-well plate by flushing the cuvette with 500 µL DMEM including 10% foetal bovine serum and 1% Penicillin/Streptomycin (Thermo Scientific). Another 500 µL including 10% foetal bovine serum and 1% penicillin/streptomycin (Thermo Scientific) was added, and cells were divided into two wells of a 24-well plate followed by a 48 h recovery under standard cell culture conditions. For determination of cytotoxicity, cells were stained with 2 ng/µL 7AAD (Santa Cruz) followed by analysis with a flow cytometer (CytoFLEX, Beckman Coulter).

### RNA Extraction and cDNA Synthesis

For RNA extraction the GeneJET RNA Purification Kit (Thermo Scientific) was used following the instructions of the manufacturer. DNAse I (Thermo Scientific), random hexamer primers (Thermo Scientific) and RevertAid Reverse Transcriptase (Thermo Scientific) for cDNA synthesis were used according to the instructions of the manufacturer.

### qPCR

Primer and probe sequences are listed in Table S2. For each reaction 25 µL containing 0.2 µM of each primer, 80 mM Tris, 20 mM (NH_4_)_2_SO_4_, 0.02% Tween20, 0.2 µM dNTP mix (Thermo Scientific), 0.005 U Taq polymerase (Agrobiogen) and 1 µL cDNA were used. Additionally, for qPCR 1 µL SYBR green to a final dilution of 1:10 (Sigma), 20 µg bovine serum albumin and 4 mM MgCl_2_ or for Taqman 0.15 µM Taqman probes and 3.5 mM MgCl_2_ were added. Measurements were taken in a Stratagene MX3000P (Agilent Technologies).

### Western Blot

SW480 cells were electroporated with 32 µM morpholino MO3. After 48-h recovery whole cell protein extracts were made with lysis buffer (20 mM HEPES pH 7.9, 400 mM NaCl, 1 mM EDTA, 0.2% NP-40, 1 mM DTT and 0.5 mM PMSF). 5 µg of the protein extracts (determined by Bradford assay) was run on an SDS page followed by semidry blotting. Membranes were blocked 1–2 h in 5% milk powder (Roth) in 1× TBS-T (10 mM Tris–Cl, pH 7.4, 150 mM NaCl, 1 mM EDTA, pH 8.0, 0.1% Tween20) on a shaker. Primary antibodies were diluted 1:500 (β-catenin sc-1496, Santa Cruz) or 1:10,000 (GAPDH sc-25778, Santa Cruz) and incubated O/N at 4 °C shaking. Secondary antibody (HRP conjugated, goat anti-rabbit sc-2004, Santa Cruz) was added in a 1:5000 dilution with 5% BSA in TBS-T. Signals were detected with ECL solution (Santa Cruz Biotechnology) and a ChemiDoc (Protein Simple) station, and quantification was done with ImageJ.

## Results

### Sensitive Detection of Antisense Effects with a Splice-Based Reporter Assay

**Fig. 1:**
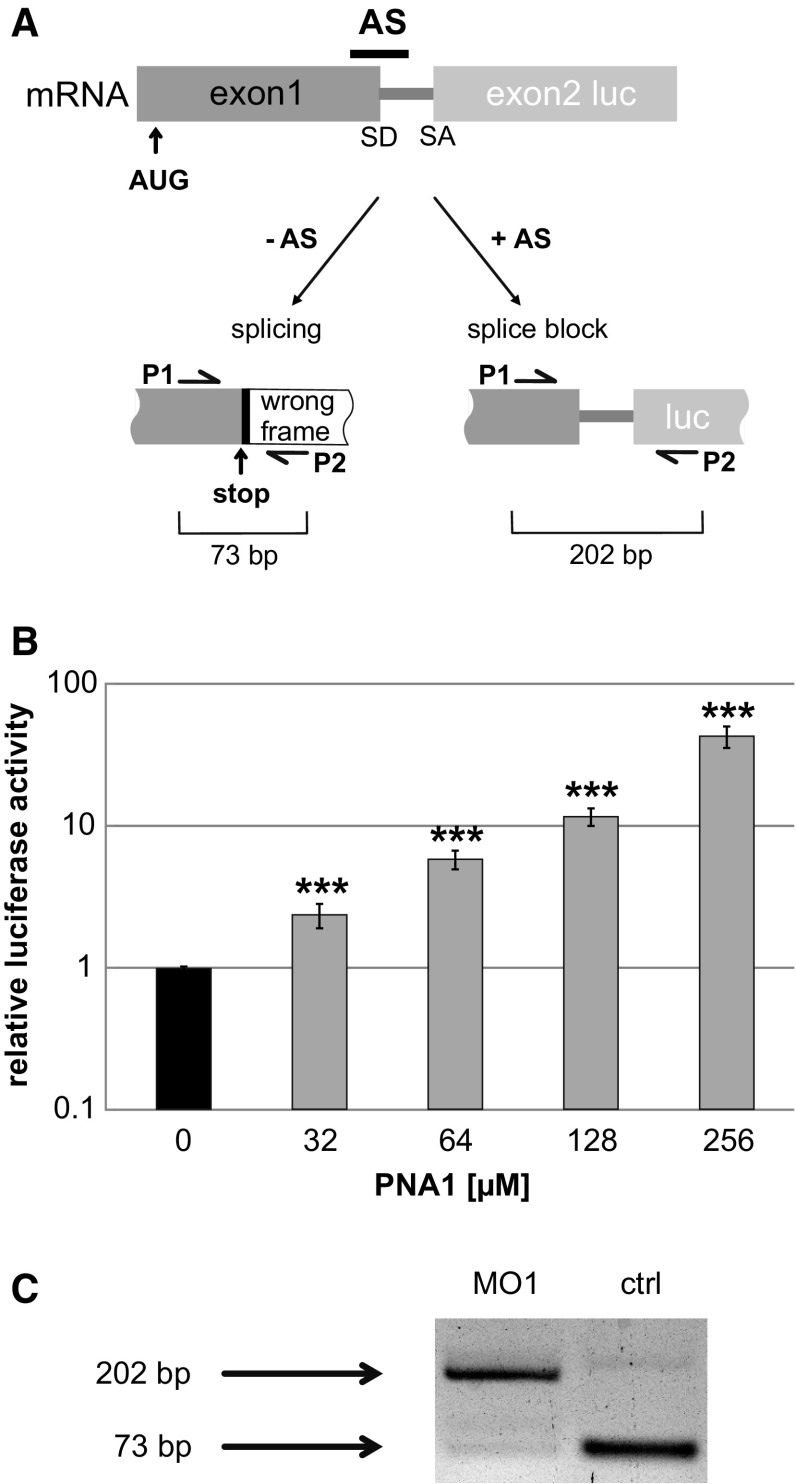
Splice-based reporter assay. **a** Schematic view of the reporter assay. AS (antisense reagent), SD (splice donor), SA (splice acceptor), P1 and P2 (primers for PCR). **b** HeLa pSplice3basis1 cells were electroporated with the indicated amount of PNA1. After 24 h luciferase activity was determined. Nluc values were divided through internal Fluc reference and normalised to control cells (0) electroporated without PNA. Data represent mean values of at least 3 independent experiments, except for 32 µM (single determination), and error bars indicate SEM. **c** PCR from cDNA of antisense-treated pSplice3basis1 cells treated with 16 µM MO1 compared to equally treated control cells without reagent (ctrl). Scraping was used for delivery, and cells were harvested after 24 h

For development of an antisense strategy targeting β-catenin, we decided to apply a splice blocking approach [42, 43]. In order to quantify the efficiency of the antisense molecules, a splice-dependant reporter assay was established. The reporter construct was designed by positioning the AUG in the first exon and an artificial intron before the second exon containing the luciferase sequence. Splicing connects exon1 with exon2 coding for luciferase, but in the wrong reading frame resulting in a non-functional protein (Fig. 1a). On the contrary, blocking of splicing by antisense molecules generates an mRNA including the non-spliced intron, which, however, contains an open reading frame directly combining those of exon 1 and 2, and resulting in a functional luciferase fusion protein. The design of the reporter construct further allows easy introduction of any splice donor (SD) of interest by using restriction sites flanking the SD. In order to test the assay, two stable cell lines containing a consensus SD sequence were generated (HeLa pSplice2basis1 and HeLa pSplice3basis1). Experiments applying the PNA oligo PNA1 (Fig. 1b; for information on the antisense oligos see Table S2) or the morpholino oligo MO1 (Fig. S1) showed a clear increase in luciferase activity (up to 49- and 13-fold, respectively). A luciferase-based internal reference was used to correct for cell number and to detect toxic effects of the antisense molecules. A concentration-dependent steady increase in luciferase activity was observed and even at the highest amounts of PNAs no drop in activity was observed, indicating no toxicity at the applied concentration range. The low cytotoxicity of the PNAs could be confirmed by flow cytometry. A concentration of 256 µM PNA1 showed no effect on the cell number (Fig. S2A) or the amount of dead cells (7AAD positive; Fig. S2B), compared to control cells electroporated without PNA. However, at 512 µM the total cell number slightly decreased and the number of dead cells increased. To confirm a splice blocking effect cDNA of HeLa pSplice3basis1 cells was analysed with PCR. The expected switch in splicing could be demonstrated using primers (P1 and P2) flanking the intron. A 73 bp product was observed for normal splicing, whereas addition of a splice blocking antisense oligo resulted in a 202 bp PCR fragment including the intron (Fig. 1a, c). The splice blocking efficiency of the morpholino oligo was strong, as indicated by the quantification of the PCR fragments resulting in a 590-fold change of the ratio upon treatment (Fig. 1c).

### Splice Blocking Strategy for β-Catenin

**Fig. 2:**
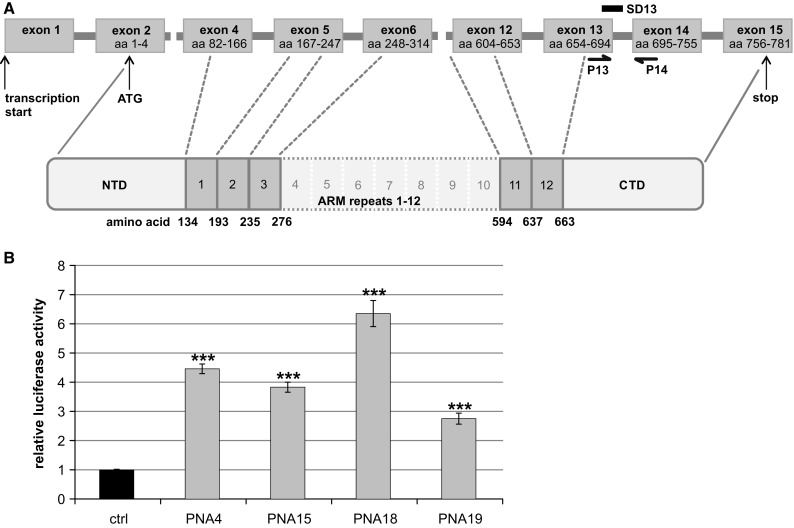
Targeting β-catenin with PNA and morpholino oligos. **a** Schematic view of the β-catenin gene including exon/intron map with positions of exon 13 SD (SD13) and splice-specific PCR primers (P13, P14), numbering of exons according to NM001904.3. The positions of the exons relative to the armadillo repeats of the protein are indicated. Abbreviations: amino acid (aa), armadillo (ARM), N-terminal domain (NTD), C-terminal domain (CTD). **b** Luciferase assay with different PNAs targeting β-catenin at the SD of exon 13. HeLa pSplice3βcat Ex13 SD cells were electroporated with 128 µM of the indicated PNAs. Nluc values were divided through the internal reference Fluc and normalised to control cells (ctrl) electroporated without PNA. Data represent mean values of at least 3 independent experiments, and error bars indicate SEM

In order to develop a strategy for targeting of β-catenin, we first tested multiple PNAs directed against the SD regions of various exons and selected the exon 13 SD for further experiments (Fig. 2a). Similar to the assay presented in Fig. 1, we established a reporter construct containing this SD and generated a stable reporter cell line (HeLa pSplice3βcat Ex13 SD). Using this assay for splice blocking, we tested 4 PNAs targeting the exon 13 SD, all with a length of 16 nucleotides and differing by a 2 nucleotide shift on their target sequences (Table S3). We observed increased luciferase levels for all 4 PNAs, and strongest luciferase induction was observed for PNA18 (Fig. 2b; 6.4-fold induction).

**Fig. 3:**
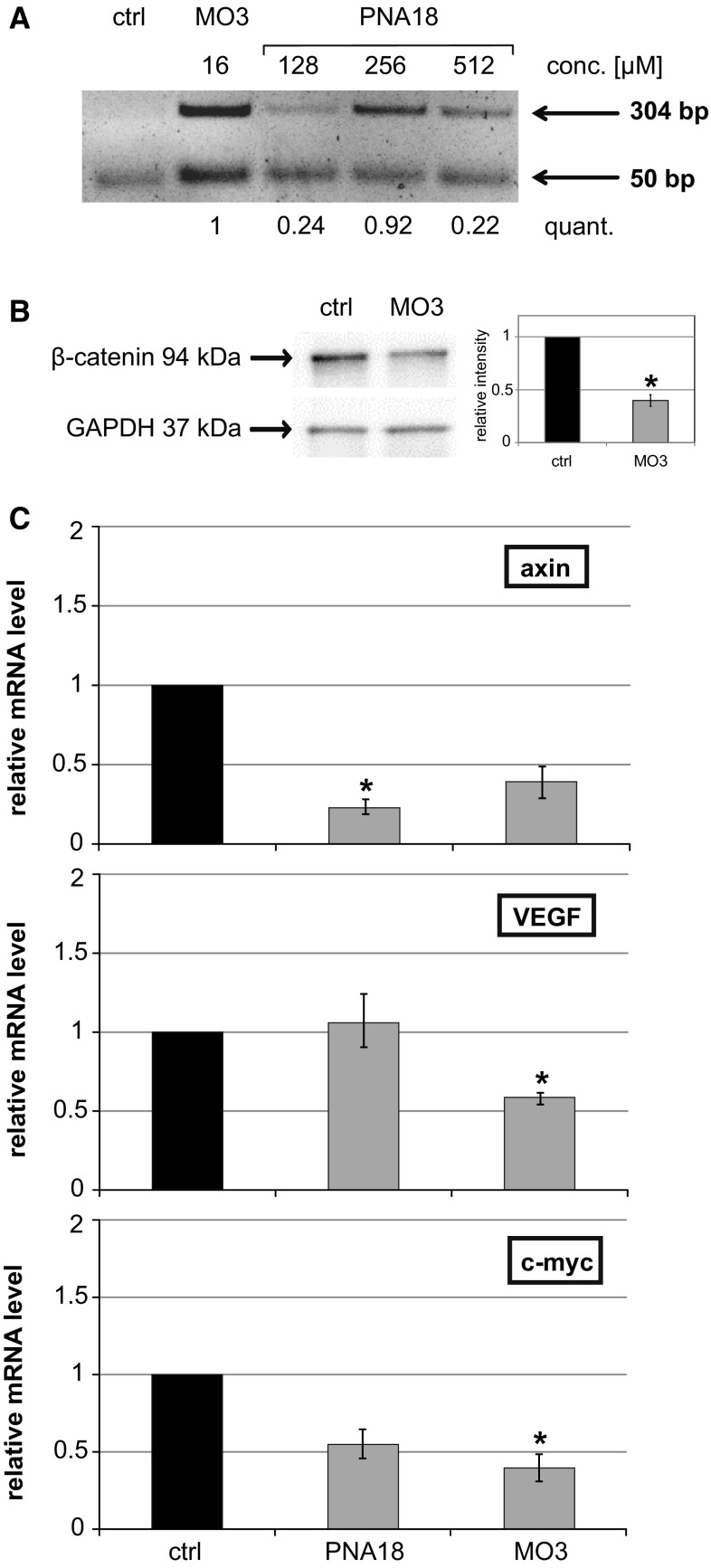
Effect of splice block at exon 13 of β-catenin in SW480 cells. **a** SW480 cells were electroporated with the indicated amounts of MO3 and PNA18. PCR from cDNA with primers P13 and P14, specific for the presence of intron 13 (304 bp band) as a result of the splice block and 50 bp resulting from normal splicing. Quantification of the band ratio normalised to MO3 is shown below (quant.). **b** Effect on β-catenin protein level in SW480 cells electroporated with 32 µM MO3, extracts prepared after 48 h. Bands for β-catenin and GAPDH are indicated. Quantification is shown for two independent experiments (resulting in a mean protein level of 40% for the MO3-treated samples compared to the control). **c** mRNA levels of the β-catenin target genes axin, VEGF and c-myc determined by qPCR normalised to GAPDH and mock-treated control cells (ctrl). SW480 cells were electroporated with MO3 (16 µM) and PNA18 (256 µM). Data represent mean values of duplicates. Error bars indicate SEM

In order to demonstrate that targeting of exon 13 SD reduced the amount of properly spliced β-catenin mRNA, a qPCR assay in SW480 cells was performed. 24 h after electroporation with the antisense molecules, β-catenin mRNA levels were detected with primers in exons 13 and 14, respectively (Table S3). PNA18 showed significant effects also in this assay (reduction to 79%; *p* = 0.0011). Contrary to the luciferase-based assay, PNAs 4, 15 and 19 did not generate a significant reduction in the qPCR assay, whereas a morpholino oligo (MO3) directed to this SD resulted in a reduction of correctly spliced β-catenin mRNA to 59% compared to the mock-treated reference (Table S3). To demonstrate that the reduced detection of β-catenin mRNA was caused by aberrant splicing, an RT-PCR experiment was performed in SW480 cells (Fig. 3a). The appearance of a 304 bp PCR product indicates the presence of intron 13 in the mRNA, whereas a 50-bp fragment appears for correctly processed mRNA, lacking the intron. Electroporation of both PNA18 and MO3 resulted in the appearance of a 304-bp fragment indicating a splice block at exon 13 SD (Fig. 3a). Furthermore, the antisense effect on β-catenin could be verified on the protein level. A Western blot with extracts harvested from SW480 cells showed a reduction of β-catenin protein to 45% compared to non-treated controls (Fig. 3b).

In order to see whether the β-catenin knock-down had an effect on established Wnt target genes, we quantified mRNA levels of axin, VEGF and c-myc in SW480 cells after PNA or morpholino treatment. In all cases we observed reduced levels upon antisense molecule application, except for PNA18 which was not effective for VEGF (Fig. 3c). In particular, PNA18 reduced the axin mRNA to 23% and that of c-myc to 55%. Similarly, MO3 reduced axin mRNA to 39%, c-myc to 40% and VEGF to 59%.

### Mammalian Two-Hybrid Analysis of β-Catenin Interactions

**Fig. 4:**
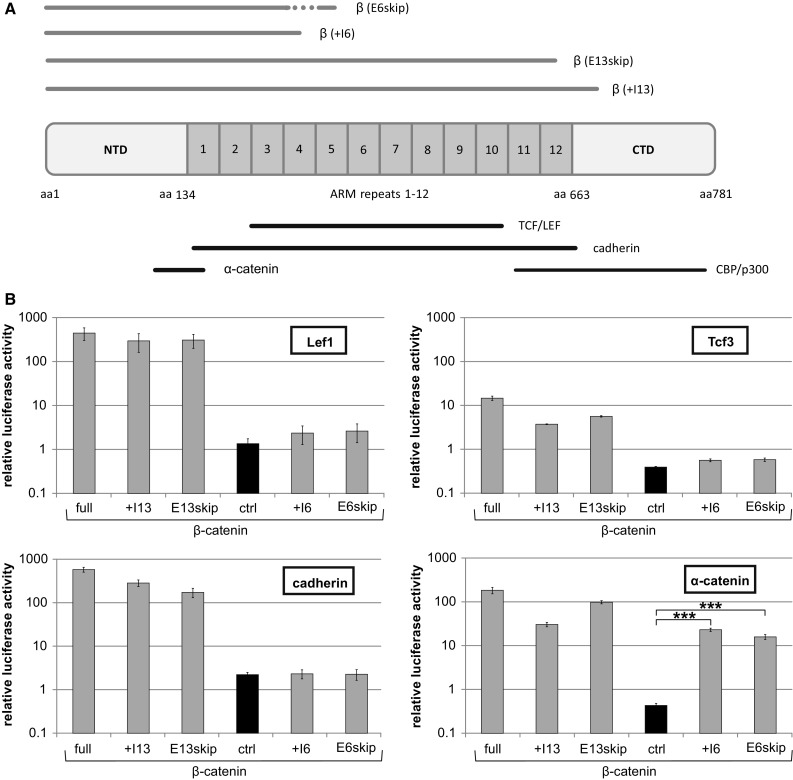
Protein–protein interactions of truncated β-catenin. **a** Schematic presentation of truncated β-catenin versions. Position of armadillo (ARM) repeats is indicated as well as the interaction sites of known binding partners [45, 46, 48]. aa (amino acids), NTD (N-terminal domain), CTD (C-terminal domain), ARM (armadillo). **b** Mammalian two-hybrid assay with the baits Lef1 (pMCLef1mZFb6), Tcf3 (pMCZFb6Tcf3), cadherin (pMCZFghe-cadherin) or β-catenin (pMCZFg alpha-catenin) together with the preys β-catenin full length (full; pMC65gDPβcat), β-catenin(+I13) (pMC65gDPβcat[1-694]), β-catenin(E13skip) (pMC65gDPβcat[1-653]), β-catenin(+I6) (pMC65gDPβcat[plusintron6]), β-catenin(E6skip) (pMC65gDPβcat[exon6skip]) and no prey (ctrl). All Fluc values were divided through the internal reference Gluc and then normalised to samples lacking bait and prey. Data represent mean values of at least 3 independent experiments, and error bars indicate SEM

The experiments so far revealed an effect of splice blocking of exon 13 SD on β-catenin mRNA, protein levels and on Wnt target genes. Splice blocking of an SD typically results in the persistence of the non-spliced intron or less often in skipping of the upstream exon. In both cases the exon 13 SD induced splice block results in a truncated β-catenin protein. The truncation would explain a lack of activity due to loss of interaction with the transcriptional co-activator CBP/p300, which depends on an intact C-terminal domain of β-catenin (Fig. 4a) [44]. To verify the reduced transactivation, we expressed a β-catenin variant truncated after exon 13 in Hela cells and measured its activity in a luciferase reporter assay (Fig. S3). Whereas full-length β-catenin activated the reporter 5.8-fold, the truncated protein resulted in luciferase values close to the basal level (1.4-fold reporter activation compared to the empty expression vector). However, β-catenin not only serves functions in transcriptional regulation, but also plays a critical role in cell adhesion. Ideally, targeting of ß-catenin should block its transcriptional activity, but retain its cell adhesion function. This function depends on protein–protein interactions with cadherin and α-catenin. Whereas the α-catenin interaction has been mapped to the N-terminal domain and armadillo repeat 1 [45], cadherin interacts with the complete central region of β-catenin (Fig. 4a) [46]. The function of β-catenin in cell adhesion could therefore be affected by truncations of the protein upon a splice block.

In order to investigate the effects of the β-catenin truncations on its interactions, we performed a mammalian two-hybrid analysis [39]. We selected blocking of two SD regions for the analysis; in addition to the SD of exon 13 (aa 654–694) we used that of exon 6 (aa 248–314) as a control, which leads to truncations lacking the majority of the armadillo repeats. In both cases we considered the presence of a non-spliced intron or skipping of the upstream exon due to the splice block. In addition, the presence of prominent cryptic splice sites in the vicinity of the SD was excluded [47]. We then generated expression constructs by PCR according to the expected splice products. For exon 6 we generated β-catenin(+I6) (intron 6 added) and β-catenin(E6skip) (exon 6 skipped) and for exon 13 β-catenin(+I13) (intron 13 added) and β-catenin(E13skip) (exon 13 skipped); the constructs were extended until the first in frame stop codon. The four truncated β-catenin versions served as prey in the mammalian two-hybrid assay and were N-terminally fused to the p65 transactivation domain. Tcf3, Lef1, cadherin and α-catenin were used as baits, fused to the DNA binding domain ZFHD [39].

For all baits interactions with full-length β-catenin as prey were clearly detectable (Fig. 4b). Also an interaction of α-catenin was detectable for all baits; however, the interactions of Tcf3 and Lef1 were clearly reduced for β-catenin(+I6) and β-catenin(E6skip), compared to that of β-catenin(+I13), β-catenin(E13skip) and β-catenin full length. Finally, the cadherin interaction was also strongly reduced for the exon 6 truncations (+I6 and E6skip), but the exon 13 truncations (+I13 and E13skip) both showed luciferase activities similar to full-length β-catenin. Therefore, truncations of β-catenin due to splice blocking at exon 13 SD retain the interactions necessary for a function in cell adhesion.

### Dominant Negative Effect of β-Catenin Truncations

**Fig. 5:**
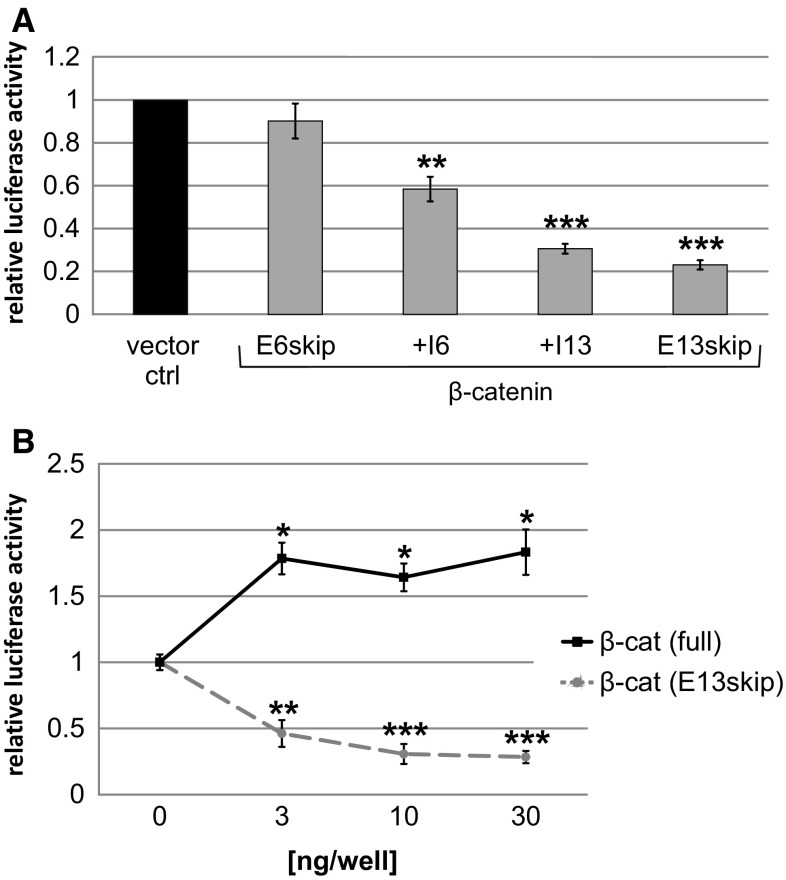
Dominant negative effect of truncated β-catenin versions. **a** 70 ng pMlucF6lefcons, 2 ng pMCGlucS and 10 ng β-catenin full length [pKCDPβcat] were co-transfected into HeLa cells with 30 ng truncated β-catenin versions: pKCDPβcat(E6skip), pKCDPβcat(+I6), pKCDPβcat(1-694)(+I13), pKCDPβcat(1-653)(E13skip) and empty pKC vector (vector ctrl). Measurements were taken 24 h after transfection. All values were normalised to samples with empty expression vector pKC. Data represent mean values of at least 3 independent experiments, and error bars indicate SEM. **b** Concentration-dependent dominant negative effect of β-catenin(E13skip) (pKCDPβcat[1-653]) compared to β-catenin full length (full; pKCDPβcat). 70 ng pMlucF6lefcons, 2 ng pMCGlucS and 10 ng pKCDPβcat were co-transfected with 3, 10 and 30 ng pKCDPβcat or pKCDPβcat(1-653) into HeLa cells. Values were normalised to controls without expression vector for β-catenin. All Fluc reporter values were divided through Gluc internal reference. Data represent mean values of at least 3 independent experiments, and error bars indicate SEM

Loss of the C-terminus is expected to result in a lack of transcriptional activity, but the mammalian two-hybrid experiments revealed binding of Tcf/Lef to the exon 13 truncations of β-catenin. This combination could be the basis for a dominant negative effect, where truncated β-catenin proteins would interfere with binding of wild-type proteins to Tcf/Lef. To test this, we transfected a β-catenin full-length expression construct together with different amounts of those for the truncated β-catenin versions. A co-transfected luciferase reporter construct containing six Tcf/Lef binding sites [49] was used for detection of Wnt pathway activity. Compared to the empty expression vector, all truncated versions resulted in reduced activity of β-catenin. However, strongest effects were seen for the two exon 13 constructs (β-catenin(+I13), reduction to 31% and β-catenin(E13skip), reduction to 23% (Fig. 5a). The most effective truncation of β-catenin(E13skip) showed effects already at low concentrations (3 ng; Fig. 5b), whereas co-transfection of full-length β-catenin had the opposite effect leading to an increase in the luciferase activity of the Wnt responsive reporter. In this experiment the activity of the full-length protein can be directly compared with the dominant negative effect of the truncated version, thus confirming the strict dependence of β-catenin on an intact C-terminus for canonical Wnt signalling.

## Discussion

### Splice-Based Reporter Assay

Alternative splicing is widely used by nature to extend the repertoire of the proteome, and aberrant splicing has been shown to be the cause for a variety of diseases [50]. Antisense molecules bind to mRNA and thus have the potential to manipulate the access of the splice machinery. Whereas most applications aim to inactivate gene function by generating non-functional proteins, also correction of aberrant splicing has been tested as a therapeutic strategy for diseases like β-Thalassaemia [43, 51], also applying reporter assays [52]. We established a luciferase-based assay which shows several advantages. The splice target sequence is integrated into an otherwise completely artificial surrounding and can easily be exchanged. The specific design of the intron allows a positive readout for the assay, meaning that successful splice blocking results in a luciferase signal above the background (Fig. 1a).

Using this reporter we developed stable cell lines for two different splice sites and in both cases observed sensitive and reliable signals for splice blocking oligos. In the case of β-catenin 4 different PNAs directed against the exon 13 SD resulted in positive signals in the luciferase assay (Fig. 2b; *p* ≤ 0.02 for all 4 PNAs). On the other hand, a qPCR-based assay showed a significant reduction of β-catenin mRNA only for PNA18 (Table S3; *p* = 0.001), whereas non-significant values were obtained for the other 3 PNAs (*p* ≥ 0.3). Main reason for this discrepancy is the error rates, which are considerably smaller for the luciferase assay (compare Fig. 2b and Table S3). Therefore, the optimisation of conditions for splice blocking works highly efficient with the presented reporter assay. Furthermore, compared to alternatives as qPCR or Western blot this assay is fast and simple.

PNAs show a number of advantageous properties compared to other antisense reagents. Due to the neutral charge of their backbone and the lack of electrostatic repulsion, they bind RNA with high affinity. Consequently, short antisense molecules (13–18 bases) are sufficient for selective binding. The small size makes them ideal for therapeutic applications, and the intracellular delivery can be further boosted using cell penetrating peptides [53], nanoparticles [54], liposomes [55] or modifications of the PNA backbone [56]. Phosphonic ester modifications of the backbone improve both the cellular uptake [57] and the knock-down efficiency [13, 14]. The phosphonic ester PNAs used in this study induced splice blocking (Fig. 1c), comparable to previous studies with PNAs [58, 59].

### Targeting β-Catenin with Splice-Based Antisense Molecules

Due to its relevance in cancer development, β-catenin has become a favoured target for nouveau cancer therapy strategies. Recent publications show decreased tumour burden within varying cancer types after targeting β-catenin with different approaches. PNAs have already been used for β-catenin knock-down in liver tumour cells [56] or antisense oligodeoxynucleotides for β-catenin targeting in xenograft mice with SW480 colon carcinoma cells [60]. Furthermore, small molecule inhibitors [61] or siRNA [62] were successfully used within xenograft mouse models. Nonetheless, rigorous targeting of β-catenin may be problematic due to the essential role of β-catenin in non-cancer related functions as cell–cell adhesion [63].

Antisense-mediated splice blocking as a strategy to reduce β-catenin levels in the cell worked in our approach and resulted in reduced levels of β-catenin mRNA, protein and consequently also of target genes (Fig. 3). A specific advantage of our splice-based antisense strategy is the generation of a dominant negative protein version. Targeting of exon 13 SD results in a C-terminally truncated protein, which lacks transactivation properties, but retains its interaction with Tcf/Lef proteins, thus competing with wild-type β-catenin (Figs. 4, 5). Most importantly, the truncation does not interfere with interactions of β-catenin with α-catenin and cadherin (Fig. 4), both critical for its function in cell adhesion. Therefore, our splice blocking antisense approach generates a truncated protein with the potential to efficiently interfere with Wnt signalling in a dominant negative manner, but at the same time retaining critical functions of β-catenin in cell adhesion. A next step would be to test this approach in an in vivo model.

## Conclusion

Herein, we present a splice-based reporter assay for accurate quantification of knock-down effects of antisense molecules. Furthermore, a strategy for targeting β-catenin next to its C-terminal transactivation domain resulted in a dominant negative protein, which efficiently blocks Wnt signalling, but retains interactions with cell adhesion molecules.

## Electronic supplementary material

Below is the link to the electronic supplementary material.

Supplementary material 1 (PDF 571 kb)
