# Supplementary material for: A Dominant Negative Antisense Approach Targeting β-Catenin
Source: Mol Biotechnol. 2018 Mar 9;60(5):339–49. doi: 10.1007/s12033-018-0058-7 (PMC5918491; doi:10.1007/s12033-018-0058-7)
Supplement: Supplementary file 1 — Supplementary material 1 (PDF 571 kb) [file 12033_2018_58_MOESM1_ESM.pdf]

Vonbrüll et al., supplementary information

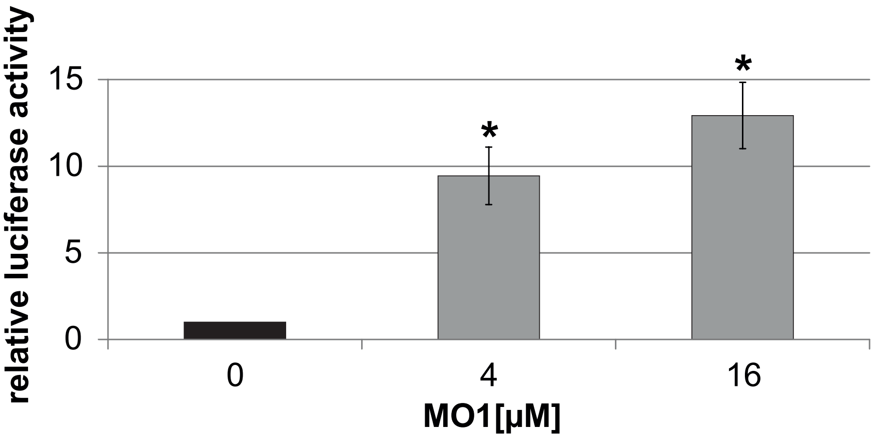

Figure S1. Splice reporter activation with morpholino oligonucleotides. Luciferase activation of HeLa pSplice2basis1 cells loaded with 0, 4 and 16 µM MO1 by scraping. Fluc values are divided by Gluc values and are normalised to samples without MO1 addition. Data show mean of at least 3 independent experiments, error bars indicate SEM.

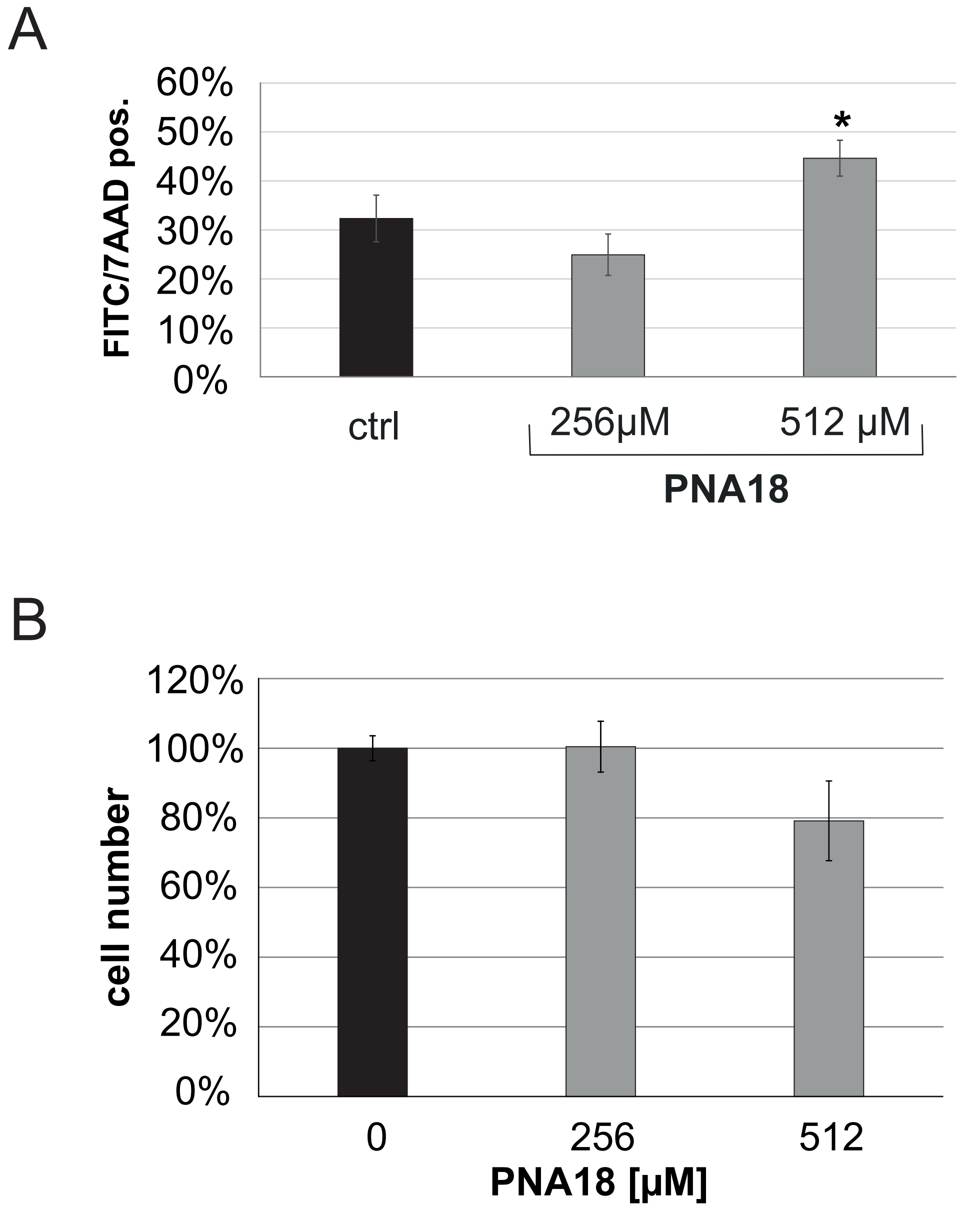

Figure S2. Toxicity of PNAs. SW480 cells were electroporated with 0.1 mg/ml FITC dextran and 0 (ctrl.), 256 and 512 µM PNA18 and analysed on the next day by flow cytometry. **A)** FITC and 7AAD positive SW480 cells. **B)** Total number of PNA18 treated cells normalised to control cells (ctrl; 0 µM). Data represent mean values of at least 3 independent experiments, error bars indicate SEM. P-value for 512 µM is 0.14.

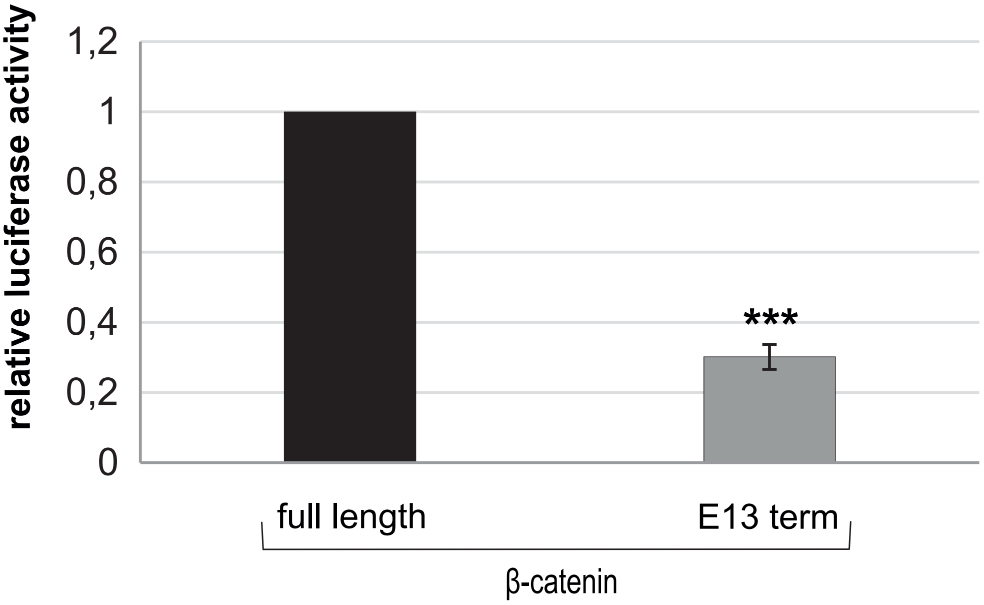

Figure S3. Transactivation of a C-terminally truncated β-catenin. 2 ng pMCGlucS, 40 ng pMlucF6lefcons, 2 ng pKCLef1 ATG and 20 ng β-catenin full length [pKCDPβcat] or E13 term [pKCDPβcat (134-663)] were co-transfected into HeLa cells. Measurements were carried out 24 h after transfection. Fluc reporter values were divided through Gluc internal reference and were then normalised to samples with empty expression vector pKC. Data represent mean values of 3 independent experiments, error bars indicate SEM.

purpose	construct	comments
**reporter assay**		
consensus SD sequence with Fluc and Gluc as reference	pSplice2 basis1	stable cell line: HeLa pSplice2 basis1
consensus SD sequence with Nluc and Fluc as reference	pSplice3basis1	stable cell line: HeLa pSplice3 basis1
β-catenin exon13 SD with Nluc and Fluc as reference	pSplice3βcat ex13SD	stable cell line: HeLa pSplice3 βcat ex13SD
**β-catenin deletion constructs**		
β-catenin full length	pKCDPβcat	full length
β-catenin (E13 term)	pKCDPβcat (134-663)	terminated after exon 13
β-catenin (+I13)	pKCDPβcat (1-694)	plus intron 13
β-catenin (E13skip)	pKCDPβcat (1-653)	exon 13 skipped
β-catenin (E6skip)	pKCDPβcat (E6skip)	exon 6 skipped
β-catenin (+I6)	pKCDPβcat (+I6)	plus intron 6
Lef reporter	pMlucF6lefcons	[49]
Lef1	pKCLef1 ATG	full length Lef1 NM_010703.4
**mammalian-two-hybrid**		
β-catenin (+I13)-p65	pMC65gDPβcat (1-694)	prey fusion with p65
β-catenin (E13skip)-p65	pMC65gDPβcat (1-653)	prey fusion with p65
β-catenin (E6skip)-p65	pMC65gDPβcat (exon6skip)	prey fusion with p65
β-catenin (+I6)-p65	pMC65gDPβcat (plus intron6)	prey fusion with p65
Lef1	pMClef1mZFb6	NM_010703.4 [39] full length bait fusion with ZFHD
Tcf3	pMCZFb6Tcf3	NM_010703.4 [39] full length bait fusion with ZFHD
E-cadherin (772-882)	pMCZFghe-cadherin	NM_001317185.1 [39] aa 772-882 bait fusion with ZFHD
α-catenin (51-268)	pMCZFg alpha-catenin	NM_001323982.1 aa 51-268 bait fusion with ZFHD
ZFHD reporter	plucF24ZF	[39]
Gaussia luciferase reference	pMCGlucS	[39]

Table S1. Plasmids

antisense oligos	target		sequence (5‘-3‘)
PNA1	SD basis		ATACTTACCTGAGAGT
PNA4	β-catenin exon 13 SD		TTCCCTACCTCATTCC
PNA15	β-catenin exon 13 SD, -2 bp		CCCTACCTCATTCCAA
PNA18	β-catenin exon 13 SD, -4 bp		CTACCTCATTCCAAGC
PNA19	β-catenin exon 13 SD, +2 bp		ATTTCCCTACCTCATT
phenylacetate-glycine	N-terminal modification of all pePNAs		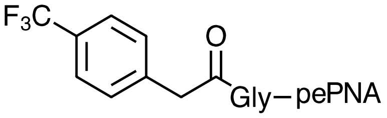
MO1	SD basis		TCGTAGCCTTGATACTTACCTGAGA
MO3	βcatenin exon 13 SD		AACTGCTCACATTTCCCTACCTCAT
**qPCR**			
target		primer	sequence (5‘-3‘)
axin (AXIN2) NM_004655.3		fwd	CATGACGGACAGCAGTGTAGATGGAA
		rev	CTCGTAGCTGCCGGAGGGCAGTAG
β-catenin (CTNNB1) NM_001904.3		fwd	CAATGGCTTGGAATGAGACTGCTGATCTT
		rev	GGTCAGTATCAAACCAGGCCAGCTGATT
c-Myc NM_002467.4		fwd	TCGGGTAGTGGAAAACCAGC
		probe	FAM-CTCCCGCGACGATGCCCCTC-BHQ1
		rev	TTCCTGTTGGTGAAGCTAACGTT
GAPDH NM_001256799.1		fwd	GGAAGGTGAAGGTCGGAGTCAA
		probe	HEX-ATTTGGTCGTATTGGGCGCCTGGTC-BHQ1
		rev	ACCAGAGTTAAAAGCAGCCCTG
VEGF-A [56]		fwd	AGGAGGAGGGCAGAATCATCA
		rev	CTCGATTGGATGGCAGTAGCT
**splice site specific**			
abbreviation	gene	primer	sequence (5‘-3‘)
P1	basis1 specific	fwd	GGATTATAAGGACGATGACGATAAGAC
P2	luciferase	rev	CTTTATGTTTTTGGCGTCTTCAGCTAG
P13	β-catenin (exon13)	fwd	AGAACAGTGCCTGGCAGCACATA
P14	β-catenin (exon14)	rev	GGCACCAATATCAAGTCCAAGATCAG

Table S2. Sequences of antisense oligos, primers and probes

	targeted position on β-cat	x-fold mRNA level	± SE
ctrl	-	1.00	0.17
PNA scrambled	-	0.99	0.16
PNA4	SD exon 13	1.00	0.14
PNA15		1.07	0.16
PNA18		0.79	0.05
PNA19		1.04	0.11
MO3		0.59	0.10

Table S3. Splice blocking of the exon 13 SD. SW480 cells were electroporated with 128 µM PNA or 8µM morpholino oligo followed by qPCR with primers in exon 13 and exon 14. Data represent mean values of at least 3 independent experiments ± SEM.
